# From Model to Crop: Roles of Macroautophagy in *Arabidopsis* and Legumes

**DOI:** 10.3390/genes16111343

**Published:** 2025-11-07

**Authors:** Lanlan Feng, Xiaowei Cui, Meng Gao, Zhenyu Wang

**Affiliations:** 1 Institute of Plant Protection, Henan Academy of Agricultural of Sciences, Zhengzhou 450002, China; fengll@link.cuhk.edu.hk (L.F.);; 2 Key Laboratory of Integrated Crop Pests Management on Crops in Southern Region of North China, Ministry of Agriculture and Rural Affairs, Zhengzhou 450002, China; 3 Henan Key Laboratory of Agricultural Pest Monitoring and Control, Zhengzhou 450002, China; 4 Biopesticide Engineering Research Center of Henan Province, Zhengzhou 450002, China

**Keywords:** *Arabidopsis thaliana*, autophagy, *ATG* genes, abiotic stress, biotic stress, leguminous plants

## Abstract

Leguminous plants are critical global crops for food security, animal feed, and ecological sustainability due to their ability to establish nitrogen-fixing symbioses with rhizobia and their high nutritional value. Autophagy, a highly conserved eukaryotic catabolic process, mediates the degradation and recycling of cytoplasmic components through the fusion of autophagosome with vacuole/lysosome and plays essential roles in plant growth, stress adaptation, and cellular homeostasis. This review systematically summarizes current knowledge of autophagy in both *Arabidopsis* and leguminous plants. We first outline the conserved molecular machinery of autophagy, focusing on core autophagy-related (*ATG*) genes in *Arabidopsis* and key legume species such as *Glycine max*, *Arachis hypogaea*, *Pisum sativum*, *Cicer arietinum*, and *Medicago truncatula*. Furthermore, the review dissects the intricate molecular regulatory networks controlling autophagy, with an emphasis on the roles of phytohormones, transcription factors, and epigenetic modifications. We then highlight the multifaceted physiological functions of autophagy in these plants. Additionally, a preliminary analysis of the *ATG8* gene family in peanut indicates that its members may be involved in seed development, biological nitrogen fixation, and drought resistance. Finally, it highlights key unresolved challenges in legume autophagy research and proposes future research directions. This review aims to provide a comprehensive theoretical framework for understanding the unique regulatory mechanisms of autophagy in legumes and to provide insights for molecular breeding aimed at developing stress-resilient, high-yielding, and high-quality legume cultivars.

## 1. Introduction

Leguminous plants (Fabaceae), the third-largest among flowing plants, are considered important crops as they provide proteins and oils for humans and farm animals around the world, contributing 27% of the world’s primary food production [1,2]. Peanut (*A. hypogaea*), soybean (*G. max*), chickpea (*C. arietinum*), common bean (*Phaseolus vulgaris*), and pea (*P. sativum*) occupy a pivotal position in global agriculture and ecological sustainability. Leguminous plants contain 2–3 times more protein than cereals like wheat, barley, corn, sorghum, and rice, with low fat content and no cholesterol, making them essential for cardiovascular health, gut health, malnutrition, and blood sugar management [3,4,5,6,7,8]. Beyond their nutritional value, legumes play a critical role in diversifying cropping system, reducing reliance on monocultures, and enhancing soil fertility through their unique symbiotic association with rhizobia-soil bacteria capable of fixing atmospheric nitrogen into ammonia, a form accessible to plants [9]. This biological nitrogen fixation (BNF) not only reduces the need for synthetic nitrogen fertilizers, lowering agricultural costs and greenhouse gas emissions from fertilizer production, but also enriches soil nitrogen levels for subsequent crops, making legumes integral to sustainable crop rotation systems [10]. Despite their significance, legume production faces challenges including abiotic stresses [11,12,13], biotic stresses [14,15,16], and yield gaps in resource-limited regions [17,18]. Understanding the biological mechanisms underlying their stress tolerance, symbiotic efficiency, and nutritional quality is thus crucial for leveraging legumes’ full potential in addressing global food security, mitigating environmental degradation, and promoting agricultural sustainability.

Autophagy is a fundamental cellular processes, literally “self-eating”, through which cells target the damaged organelles or proteins to the vacuole/lysosome for degradation and potential reuse of the resulting molecules. In eukaryotes, autophagic routes have been classified into three major types: chaperone-mediated autophagy [19], microautophagy [20], and macroautophagy [21]. In plant, macroautophagy (hereafter referred to as autophagy) is a process by which large cytoplasmic cargo such as protein or pigment aggregates, molecular complex, and damaged organelles is delivered to the vacuole. This mechanism enables plant survival during challenges such as nutrient deprivation, abiotic stress, and pathogen infection [22,23]. Conserved autophagosome biogenesis process includes over 20 autophagy-related (*ATG*) genes in *Arabidopsis*. Based on their interactions, the encoded ATG proteins can be divided into 5 core subgroups: (1) the ATG1/ATG13 protein kinase complex, critical for autophagy initiation, nutritional signal sensing, the recruitment of downstream ATG proteins, and the formation of autophagosome [24]; (2) ATG9 vesicles and the ATG2-ATG18 complex, required for phagophore expansion [25]; (3) the class III phosphatidylinositol 3-kinase (PI3K) complex, vital for autophagy and endosomal maturation [26]; (4) the ATG12-ATG5 conjugation system, indispensable for the incorporation of ATG8-PE into the expanding phagophore [27]; (5) the ATG8 conjugation system, essential for orchestrating membrane formation and selective autophagy [28].

In *Arabidopsis*, ATG6 is essential for restricting the pathogen-induced cell death response [29]. ATG5 plays essential role in leaves for nitrogen supply and in seeds for the establishment of carbon and nitrogen reserves [30]. All canonical *atg* mutants exhibit phenotypes characterized by accelerated leaf senescence and hypersensitivity to carbon and nitrogen limitation [31]. In rice (*Oryza sativa*), *Osatg7-1*, an autophagy-disrupted rice mutant, showed reduced biomass production and nitrogen use efficiency compared with the wild type [32]. In addition, the seeds of *Osatg7-1* are smaller and show chalky appearance and lower starch content in the endosperm at the mature stage under normal growth condition [33]. Overexpression of the rice gene *OsATG8b* enhances tolerance to nitrogen starvation, while simultaneously improving yield and nitrogen use efficiency in *Arabidopsis* [34]. The *OsATG9* genes counteract autophagy to balance growth and drought tolerance in rice [35]. In maize (*Zea mays*), *ZmATG3*-overexpressing *Arabidopsis* exhibit higher osmotic/salinity tolerance and upregulated expression of other *AtATGs* under stress, indicating *ZmATG3* overexpression improves tolerance to multiple abiotic stresses [36]. *ZmATG8c* enhances maize thermotolerance during both vegetative and reproductive growth stages by increasing autophagosome production, elevating ATG8 levels, and reducing the accumulation of ubiquitinated protein aggregates, thereby improving seedling survival and grain yield under heat stress [37]. *ZmATG18a* exerts a positive regulatory role in maize responses to drought stress: specifically, a gain-of-stop EMS mutant of *ZmATG18a* displayed heightened sensitivity to drought, whereas a Mutator transposon-induced mutant with upregulated *ZmATG18a* expression exhibited enhanced drought tolerance [38].

Although ATG genes have been identified and characterized in *Arabidopsis* and Poaceae plants, research on autophagy in Fabaceae plants remains relatively limited and requires systematic integration. The uniqueness and scientific value of autophagy research in legumes stem from their distinctive biological traits, particularly the evolutionarily conserved yet specialized symbiotic nitrogen-fixing relationship with rhizobia, which sets them apart from other plant families. For instance, autophagy in legumes mediates not only canonical functions like cellular homeostasis under abiotic stresses but also orchestrates nodule development, senescence, and the dynamic allocation of fixed nitrogen [39,40,41]. This specialized context highlights legume-specific autophagy mechanisms, including the regulation of *ATG* gene expression during nodule maturation and the selective degradation of symbiosis-associated proteins, which collectively fine-tune rhizobial colonization.

## 2. Molecular Mechanisms of Autophagy in *Arabidopsis* and Leguminous Plants

In response to rapidly fluctuating environments, eukaryotes have evolved diverse mechanisms to preserve cellular homeostasis, facilitate nutrient recycling, and conduct quality control of cellular components under both basal and stressful conditions. Autophagy represents a highly conserved catabolic process that plays vital roles in plant growth, development, and stress adaptation [42]. Notably, phytohormones also serve as central regulators of many of these physiological and stress-responsive processes [43]. Accumulating experimental evidence underscores an intimate crosstalk between autophagy and phytohormone signaling cascades, and the underpinning molecular mechanisms governing these interactive pathways have started to be unraveled. In additon, the nuclear events involved in the regulation of plant autophagy have been emerged [44]. Here, we review recent advances in our understanding of how hormones, transcription factors, and epigenetic modifiers regulate the activity of autophagy in *Arabidopsis* and legumes.

### 2.1. Phytohormones and Autophagy in Arabidopsis and Legumes

In *Arabidopsis*, in the presence of brassinosteroids (BRs), enhanced BR signaling abrogates the phosphorylation of regulatory-associated protein of TOR 1B (RAPTOR1B) and brassinosteroid insensitive 1-EMS-suppressor 1 (BES1) by the glycogen synthase kinase 3 (GSK3)-like kinase brassinosteroid-insensitive 2 (BIN2). This inhibition of BIN2-mediated phosphorylation promotes target of rapamycin (TOR) kinase activity, which in turn downregulates autophagic flux. Conversely, under BR-deficient conditions, BIN2 retains its kinase activity to phosphorylate both RAPTOR1B and BES1. The consequent attenuation of BR signaling ultimately results in the induction of autophagy [45]. During leaf senescence and pathogen ingress, salicylic acid (SA) signaling is amplified through the activation of SA biosynthetic pathways, thereby establishing an amplification loop dependent on ENHANCED DISEASE SUSCEPTIBILITY 1 (EDS1). In parallel, this SA-driven signaling pathway elicits autophagy via NONEXPRESSOR OF PR GENES 1 (NPR1); notably, autophagy functions as a negative feedback loop to modulate SA signaling, which in turn constrains the progression of senescence and pathogen-triggered chlorotic cell death [46]. In *Arabidopsis*, ethylene-induced autophagy acts as a protective mechanism that enhances root cell survival in early waterlogging stress by targeting compromised mitochondria for degradation, which in turn limits reactive oxygen species (ROS) production under concomitant oxidative stress [47]. An elevation in intracellular abscisic acid (ABA) levels transiently reduces the persulfidation status of the ATG4 protein pool activating the protease activity of the enzyme and the processing of ATG8 that can be further lipidated to progress autophagy [48]. The phytohormone cytokinin and autophagy interact through a mechanism where selective autophagy, with EXO70D proteins acting as receptors, degrades type-A ARRs (negative regulators of cytokinin signaling) in an ATG5-dependent and phosphorylation-promoted manner to modulate plant sensitivity to cytokinin, while this autophagy–cytokinin interplay also contributes to plant survival under stress condition [49]. Jasmonic acid (JA) promotes chromatin remodeling to alleviate the repression of MYC transcription factors, thereby enhancing chromatin accessibility for downstream targets such as NAC DOMAIN-CONTAINING PROTEIN102 (ANAC102). Before abscission, ANAC102 specifically accumulates at the petal base, where it upregulates ATG gene expression. This induction triggers reactive oxygen species (ROS) accumulation, cell death, and developmentally programmed autophagy, collectively contributing to petal abscission [50].

**Figure 1:**
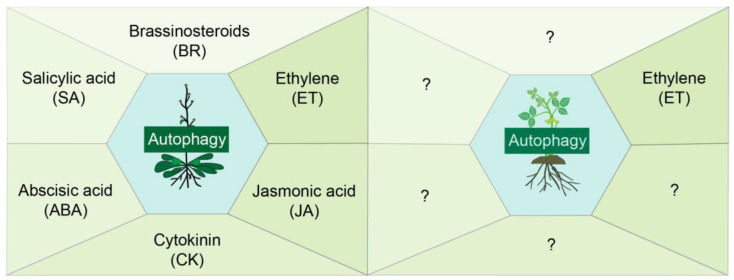
Regulation of autophagy by diverse phytohormones in *Arabidopsis* and leguminous plants. In *Arabidopsis*, BR can regulate autophagy both positively and negatively, whereas SA, ET, ABA, JA, and CK activates autophagy during biotic or abiotic stress conditions. In leguminous plants, ET activates autophagy under starvation stress conditon. The figure was prepared using Adobe Illustrator CC 2017 software package. The question marks indicate that the role of the corresponding phytohormone in the regulation of autophagy is currently unknown in legumes.

However, research on phytohormone-autophagy crosstalk in leguminous plants remains remarkably scarce; to date, only one study has been reported that the induction of GmATG8i in soybean plants under starvation stress is mediated by the ethylene signal [51]. Deciphering the phytohormonal regulation of autophagy holds the key to understanding how leguminous plants manage the critical trade-off between growth and stress adaptation (Figure 1).

### 2.2. Transcription Factors and Epigenetic Regulation of Autophagy in Arabidopsis and Legumes

In *Arabidopsis*, TGA9 (TGACG (TGA) motif-binding protein 9) is a confirmed positive regulator of autophagy that binds the promoters of *ATG8* genes [52]. Furthermore, PHYTOCHROME-INTERACTING FACTOR4 (PIF4) and PIF5 directly bind to the promoters of core ATG genes, including ATG5, ATG12a/b, ATG8a/e/f/g, inducing their expression, and influencing nutrient starvation induced senescence, a hallmark autophagy ralated process [53]. ATG18a, a core component of the autophagic machinery in *Arabidopsis*, physically interacts with WRKY33 which is a transcription factor essential for mediating resistance against necrotrophic pathogens. Notably, infection by the necrotrophic fungal pathogen *Botrytis cinerea* elicits the transcriptional upregulation of *ATG* genes and promotes autophagosome biogenesis in *Arabidopsis*. Importantly, the *B. cinerea*-triggered induction of *ATG18a* expression and autophagic activity was significantly impaired in the *wrky33* loss-of-function mutant, which exhibits heightened susceptibility to necrotrophic pathogens [54]. Yang et al. demonstrated that autophagy displays a robust circadian rhythm in *Arabidopsis* under both light/dark cycles (LD) and constant light (LL) conditions. Furthermore, they showed that under LD conditions, the transcription factor LUX ARRHYTHMO (LUX) directly binds to the promoters of core autophagy genes *ATG2*, *ATG8a*, and *ATG11* to repress their expression. Consequently, the lux mutant exhibits a disrupted autophagy rhythm in LL and an increased rhythm amplitude in LD, due to the loss of this nocturnal repression [55]. The flowering transition factor SUPPRESSOR OF OVEREXPRESSION OF CONSTANS1 (SOC1, a MADS-box protein) acts as a transcriptional repressor of autophagy. Mechanistically, SOC1 binds directly to the CArG box elements in the promoters of key autophagy genes, including ATG4b, ATG7, and ATG18c, to suppress their expression [56]. The NAC (for NO APICAL MERISTEM/ARABIDOPSIS TRANSCRIPTION ACTIVATION FACTOR/CUP-SHAPED COTYLEDON) transcription factor ATAF1 (*Arabidopsis* Transcription Activation Factor1) regulates the expression of multiple autophagy-related genes. Overexpression of ATAF1 induces transcriptomic changes, upregulating 11 ATG genes in a pattern that mimics carbon starvation in wild-type plants. Conversely, ATAF1 depletion represses these genes. These findings suggest that ATAF1 acts as a key regulator of autophagy induction during carbon starvation [44]. RNA sequencing analysis revealed that elevated expression of the NAC domain transcription factor ANAC017 results in the upregulation of genes associated with mitochondrial stress, cell death, and autophagy under non-stress conditions, while also triggering extensive repression of chloroplast function and promoting a leaf senescence signature [57]. The transcription factor ELONGATED HYPOCOTYL 5 (HY5) recruits HISTONE DEACETYLASE 9 (HDA9) to the loci of *ATG5* and *ATG8e*, thereby repressing their expression through histone deacetylation at H3K9 and H3K27. Consistently, HY5 deficiency leads to elevated *ATG* gene expression, enhanced autophagy activity, and, consequently, improved resistance to prolonged darkness and nitrogen starvation [58].

The function of TGACG-Binding (TGA) transcription factors, long recognized for defense roles in *Arabidopsis*, may extend beyond this, as evidenced by the discovery of two legume-specific GmTGA proteins in soybean, which are characterized by unique autophagy-associated sequence motifs [59]. Research investigating transcription factor- and epigenetic-mediated regulation of autophagy in leguminous plants remains notably scarce, highlighting an urgent need for in-depth studies in this underrepresented area.

## 3. Biological Functions of Autophagy in *Arabidopsis* and Leguminous Plants

Fueled by global warming and climate change, abiotic stressors including drought, extreme temperatures, and flooding are becoming more frequent and severe, posing a growing threat to agricultural yields and food security worldwide [60]. Moreover, climate change is reshaping the composition and dynamics of insect and pathogen communities, further exacerbating yield losses on a global scale. Agriculture is also constrained by the growing accumulation of anthropogenic pollutants and by climate-driven disruptions to soil microbiomes, which can impair ecosystem functioning and nutrient cycling [61]. A concentrated effort is needed to study how stress is affecting crops.

### 3.1. Biological Functions of Autophagy in Arabidopsis

**Figure 2:**
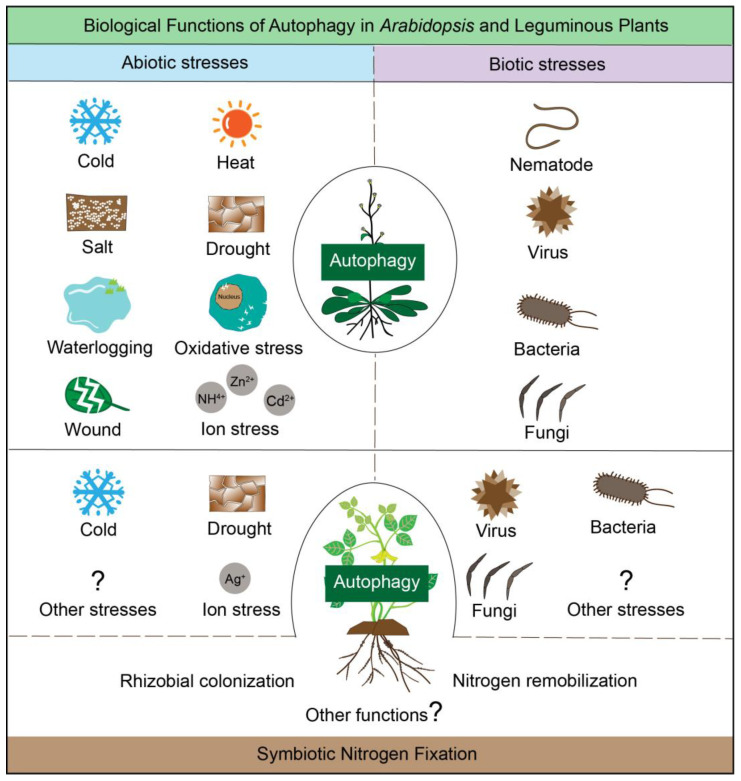
Biological functions of autophagy in *Arabidopsis* and leguminous plants. Autophagy is involved in regulating plant responses to various abiotic and biotic stresses. Additionally, in leguminous plants, autophagy further participates in the process of biological nitrogen fixation—a function unique to this plant family. However, compared with *Arabidopsis*, research investigating the role of autophagy in leguminous plants’ defense against abiotic and biotic stresses remains remarkably scarce, highlighting an urgent need for more in-depth studies in this area.

Autophagy serves as a key regulatory mechanism for orchestrating adaptation to diverse abiotic and biotic stresses in *Arabidopsis*, specifically including nitrogen starvation [62], cold damage [63], heat stress [64,65,66], salt stress [67], drought [68,69], waterlogging stress [70], oxidative stress [71], wounding [72], cadmium stress [73], ammonium stress [74], zinc stress [75], nematode [76], virus, such as Turnip mosaic virus [77] and Cauliflower mosaic virus [78], bacteria, such as *Pseudomonas syringae* [79], and pathogen, such as *Alternaria brassicicola* [80,81], *B. cinerea* [54], *Diplodia cupreessi* [82], *Golovinomyces cichoracearum* [83,84], *Sclerotinia sclerotiorum* [85], *Verticillium dahliae* [86]. Research on autophagy in leguminous plants remains relatively scarce. Therefore, given legumes’ global importance in food security and sustainable agriculture, deciphering their autophagic regulation could inform strategies to enhance stress tolerance, optimize nitrogen use efficiency, and prolong nodule functionality, thereby addressing pressing challenges in crop improvement and ecological sustainability. Thus, legume autophagy research bridges fundamental cell biology with applied agricultural science, offering unique insights that transcend species-specific studies and advance the broader field of plant stress physiology and symbiosis (Figure 2).

### 3.2. Autophagy in Abiotic Stress Tolerance of Legumes

A set of 39 autophagy-related genes (ATGs) identified in *M. truncatula* showed significant upregulation during seed development and under drought stress, suggesting an important role for autophagy in these critical biological processes [87]. In *Medicago sativa*, cold stress triggers the transcriptional activation of *MsATG13*, which functions to integrate autophagic flux with the regulation of antioxidant levels, thereby mediating stress adaptation [88]. A *M. truncatula* dehydrin named MtCAS31 (Cold acclimation-specific 31) is a positive regulator of drought response and plays an important role in autophagic degradation of proteins, such as MtPIP2;7, which functions as a negative regulator of drought response. Under drought stress, MtCAS31 promotes the autophagic degradation of MtPIP2;7 and decreases root hydraulic conductivity, thus reducing water loss and enhancing drought tolerance [89]. In *P. vulgaris*, *PvATG8c* participated in virus-induced drought tolerance via autophagy mediated the degradation of PvERD15 (Early Responsive to Dehydration 15, which positively regulates stomatal aperture) [90]. Transmission electron microscopy analysis demonstrated that drought stress in *Caragana korshinskii* triggers autophagosome formation and chloroplast degradation, with the intensity of autophagic activity and cytoplasmic turnover increasing alongside stress severity, accompanied by a rise in sucrose levels—pointing to a coordinated physiological response [91]. In the response of *Trifolium pratense* L. (red clover) to Ag^+^ stress, autophagy is annotated as one of the stress-responsive pathways via Kyoto Encyclopedia of Genes and Genomes (KEGG) analysis, suggesting it may play a role in mediating the plant’s Ag^+^ tolerance [92].

### 3.3. Autophagy in Biotic Stress Defense of Legumes

In soybean, silencing of *GmATG2* impairs autophagic degradation, accelerates dark-induced senescence, and activates immune responses, as evidenced by elevated levels of reactive oxygen species (ROS) and salicylic acid (SA), upregulated expression of *pathogenesis-related gene 1* (*PR1*), and enhanced resistance to *Pseudomonas syringae pv. glycinnea* [39]. *GmATG7* silencing impaired autophagy, accelerated dark-induced leaf senescence, induced H_2_O_2_ and SA accumulation to activate immunity, and enhanced resistance to *Pseudomonas syringae pv. glycinnea* and *Soybeann mosaic virus* [93]. The glycine-rich plasma membranne PSS1 protein exhibited enhanced resistance to *Fusarium virguliforme*, the pathogen that causes sudden death syndrome, in transgenic soybean plants, and autophagy is proposed to be involved in this associated process [94]. In addition, in the symbiosis between *M. truncatula* and arbuscular mycorrhizal (AM) fungi, autophagy-related genes are expressed in AM fungal arbuscules, suggesting that autophagy may contribute to sustaining arbuscule function during this mutualistic interaction [95].

### 3.4. Autophagy in Seed Development and Symbiotic Nitrogen Fixation of Legumes

*GmATG8c* constitutively expression in soybean callus enhanced the cells’ nitrogen starvation tolerance and accelerated callus growth, while transgenic *Arabidopsis* overexpressing *GmATG8c* exhibited better performance under prolonged nitrogen and carbon starvation, faster growth, earlier bolting, larger primary and axillary inflorescences, and a 12.9% higher average seed yield than wild-type plants under optimal growth conditions, leading to the conclusion that *GmATG8c* is a promising candidate for breeding crops with improved nitrogen use efficiency (NUE) and yield [96]. GmATG8c facilitates the redistribution of nitrogen from vegetative tissues to developing seeds, thereby positioning it as a potential initiator of leaf senescence [97]. In white lupin (*Lupinus albus* L.) and Andean lupin (*Lupinus mutabilis* Sweet), autophagy is constitutively active from the earliest stages of growth, including within embryonic seed organs. This process is markedly upregulated under nutrient deficit or starvation, thereby promoting survival under adverse conditions [40,98]. In *P. vulgaris* and *G. max*, 32 and 61 core *ATG* genes were identified. Notably, most *ATG* genes in *Phaseolus* exhibit nitrate-responsive expression patterns and are differentially regulated during rhizobial and mycorrhizal symbioses, suggesting their potential functional involvement in these symbiotic interactions [99,100,101]. In silico expression analysis, *ATG* genes of all groups were expressed in the root nodule of *M. truncatula*, this link between autophagy and symbiosis needs more attention [102]. Further evidence indicates that the interaction between MtNAD1 and MtATG8 functions to recruit the autophagy machinery for the targeted degradation of immunity-related proteins in *M. truncatula* nodules. This targeted proteolysis enables successful rhizobial colonization and the subsequent establishment of symbiosis [41]. Moreover, root- and nodule-predominant myotubularin phosphatase (*M. truncatula*, MtMP) promotes autophagy via dephosphorylating autophagosomal phosphatidylinositol 3-phosphate (PtdIns3P); this MtMP-mediated autophagy (likely mitophagy in the nodule infection zone) supports symbiotic nitrogen fixation by sustaining nitrogenase activity and supplying carbon skeletons/nitrogen for bacteroid development and infected cell reprogramming, representing the first report of myotubularin phosphatase in plant autophagy [103]. In pea (*P. sativum* L.), autophagy-related genes are transcriptionally upregulated in leaves at the late stage of early seed development (27 days after flowering) and thus likely participate in nutrient recycling from leaves to support seed protein accumulation, a key determinant of pea protein yields [104]. Nitrogen constitutes one of the indispensable plant nutrients and serves as a key constraint on crop productivity. To fulfill the demands of sustainable agriculture, there is a need to maximize biological nitrogen fixation in legumes [105]. To date, overexpression of rice *OsATG8b* and *OsATG8c* was found to enhance autophagic activity, leading to a significant improvement in nitrogen remobilization efficiency (NRE) from vegetative tissues to seeds, as evidenced by 15N-labeling experiments [106,107]. The role of autophagy in regulating nitrogen remobilization has also been established in a range of other species, including maize [108], tomato [109], tea plants [110], and apple [111]. Therefore, elucidating the role of autophagy in leguminous plants, particularly its functions in nitrogen remobilization and symbiotic nitrogen fixation, represents a pivotal strategy for developing crops that require less fertilizer yet maintain high yield and improved nutritional quality [112].

## 4. Genome-Wide Identification of ATG8 Gene Family in Peanut

**Figure 3:**
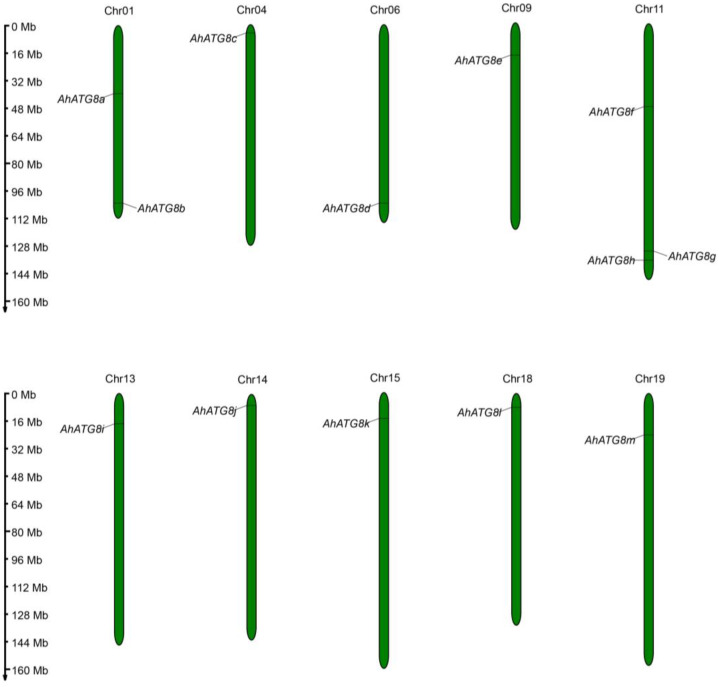
The chromosome distribution of *AhATG8* family genes in *A. hypogaea*. The *AhATG8s* sequences data derived from the PeanutBase (13 October 2025, https://www.peanutbase.org, *A. hypogaea* Tifrunner *Tifrunner.gnm1.ann1.CCJH* Annotations) with default parameters. This image was generated with MG2C v2.1 (http://mg2c.iask.in/mg2c_v2.1/index.html, accessed on 15 October 2025).

Peanut (*A. hypogaea* L.) is a valuable legume crop worldwide, possessing a favorable nutritional profile notably rich in proteins, healthy lipids, vitamins, and minerals, which contributes to its global significance [113]. However, the susceptibility to diverse abiotic and biotic stresses poses a major constraint to peanut productivity, leading to significant economic losses [114]. Beyond its well-established role in nutrient starvation, a growing body of evidence demonstrates that autophagy is also induced as an adaptive cellular response to diverse environmental challenges, including abiotic stresses such as drought, salinity, and extreme temperatures, as well as biotic stresses [115,116]. ATG8 proteins play a central role in plant autophagy by not only promoting autophagosome formation but also interacting with various adaptor/receptor proteins, thereby recruiting specific cargo for degradation through selective autophagy [117]. Nine ATG8 isoforms have identified in *Arabidopsis*, nine in wheat [118], five in maize [119], seven in rice [120], seven in potato [121], and eleven in soybean [96]. To understand the role of autophagy response to abiotic and biotic stresses in peanut, a total of 13 members (named *AhATG8a* to *AhATG8m*) of the *ATG8* gene family were identified in peanut through bioinformatics analysis (Figure 3 and Table 1).

**Figure 4:**
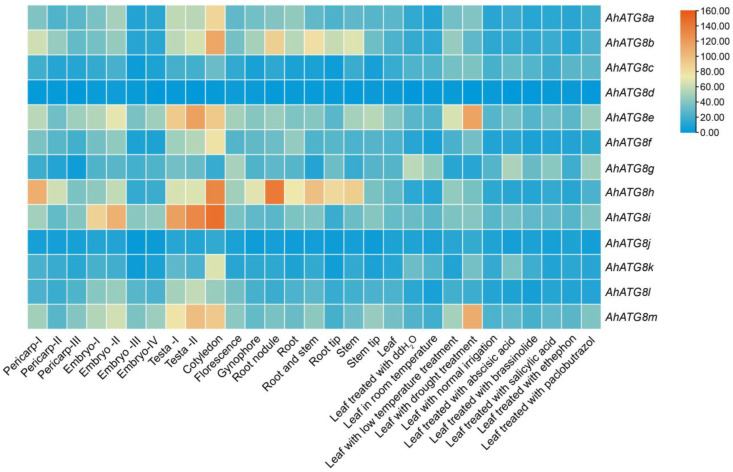
Expression profile of *AhATGs* genes of *A. hypogaea* in different tissues and treatments. Transcriptome data regarding the expression levels (Normalized expression values in FPKM) of all *AhATG8* members across 29 different peanut tissues and treatments were obtained from the PeanutGenomeResource website (http://peanutgr.fafu.edu.cn/Transcriptome.php#results, accessed on 15 October 2025). The heatmap was generated using the HeatMap function in TBtools v2.152 software, log scale (base = 2 and logwith = 1).

In addition, gene expression pattern analysis revealed that *AhATG8a*, *AhATG8b*, *AhATG8e*, *AhATG8f*, *AhATG8h*, *AhATG8l*, *AhATG8m*, and particularly *AhATG8i*, exhibited high expression levels in the testa and cotyledons. Several genes encoding the core autophagy machinery are induced during seed development [122]. Research has shown that AtATG8b is involved in microlipophagy-mediated lipid droplet (LD) degradation in *Arabidopsis*. CALEOSIN family proteins CLO1, CLO2, and CLO3, as structural LD proteins, interact with AtATG8b via their putative ATG8-interacting motifs (AIMs). Specifically, the AIM preceding the proline knot in CLO1 is critical for its interaction with AtATG8b, indicating that these ATG8b-caleosins mediate the recruitment of LDs to the vacuole for degradation during seed germination [123]. The *AhATG8s* genes, which are highly expressed in the testa and cotyledons, may regulate lipid degradation via the autophagic pathway to participate in seed development. Furthermore, Crop legumes, including chickpea, common bean, cowpea, peanut, pigeonpea, and soybean, serve as vital nutritional sources and account for substantial biological nitrogen fixation in agriculture, with the fixed nitrogen exceeding 20 million tons annually [124]. *AhATG8b* and *AhATG8h* showed high expression in root nodules, implying that these two genes might be associated with nodule formation or biological nitrogen fixation processes. Under drought stress, the expression levels of *AhATG8e* and *AhATG8m* were upregulated, which demonstrates their role in responding to drought stress (Figure 4).

## 5. Perspectives

Leguminous plants exhibit extensive species diversity and a wide distribution, serving as high-quality food sources for humans and animals. Compared with *Arabidopsis*, research on autophagy in legumes remains relatively scarce. Therefore, conducting studies on autophagy in legumes can help identify potential stress resistance pathways and facilitate the breeding of new stress-tolerant legume varieties. Additionally, ATGs are involved in nodulation of leguminous plants, which will provide novel candidate genes for enhancing nitrogen-fixation capacity in legumes. Expression analysis of the *AhATG8* gene family in peanut revealed that *AhATG8b* and *AhATG8h* were highly expressed in root nodules, indicating that these two genes may be involved in the biological nitrogen fixation process of peanut. Therefore, we could generate stable transgenic peanut plants overexpressing *AhATG8b* or *AhATG8h*. Conversely, use CRISPR-Cas9 to create knockout mutants for these genes. Phenotypic analysis should focus on nodulation (number, size, morphology), nitrogen fixation efficiency (measured by acetylene reduction assay), and overall plant growth under nitrogen-limiting conditions. Furthermore, *AhATG8e* and *AhATG8m* exhibited high expression levels under drought treatment condition, indicating that *AhATG8e* and *AhATG8m* participated in the drought response of peanut. Similarly, develop overexpression lines and knockout mutants for AhATG8e and AhATG8m. These plants should be subjected to controlled drought stress, and their performance should be evaluated based on water use efficiency, photosynthetic parameters, oxidative stress markers, and survival rates, alongside monitoring autophagic activity. Further investigations into the precise mechanisms governing the regulation, subcellular localization, protein interaction screening, and functional roles of peanut *AhATG8* genes are essential, particularly regarding whether *AhATG8s* can activate autophagy to enhance the response to both abiotic and biotic stresses.

## Figures and Tables

**Table 1:** The *AhATG8s* gene family members of *A. hypogaea*.

Member	Locus	Chromosomal Location	Gene Length/bp	Protein Length/bp
*AhATG8a*	*arahy.Tifrunner.gnm1.ann1.JG4KI1.1*	Chr01:39903752-39907161	3410	156
*AhATG8b*	*arahy.Tifrunner.gnm1.ann1.EXRI64.1*	Chr01:103592801-103594956	2156	119
*AhATG8c*	*arahy.Tifrunner.gnm1.ann1.IDQ1YW.1*	Chr04:4930217-4936705	6489	230
*AhATG8d*	*arahy.Tifrunner.gnm1.ann1.6U9WS0.1*	Chr06:103978667-103981122	2456	125
*AhATG8e*	*arahy.Tifrunner.gnm1.ann1.63V2PN.1*	Chr09:18979110-18985083	5974	118
*AhATG8f*	*arahy.Tifrunner.gnm1.ann1.0A7WSD.1*	Chr11:48330080-48333436	3357	122
*AhATG8g*	*arahy.Tifrunner.gnm1.ann1.SY21CD.1*	Chr11:132703325-132704747	1423	123
*AhATG8h*	*arahy.Tifrunner.gnm1.ann1.8SMT51.1*	Chr11:137923012-137926868	3857	177
*AhATG8i*	*arahy.Tifrunner.gnm1.ann1.X3Q019.1*	Chr13:17577237-17580265	3029	120
*AhATG8j*	*arahy.Tifrunner.gnm1.ann1.CQM6UK.1*	Chr14:6182864-6185138	2275	119
*AhATG8k*	*arahy.Tifrunner.gnm1.ann1.E4CLSM.1*	Chr15:15136078-15138924	2847	114
*AhATG8l*	*arahy.Tifrunner.gnm1.ann1.ZFW017.1*	Chr18:7807375-7809476	2102	120
*AhATG8m*	*arahy.Tifrunner.gnm1.ann1.HJA00B.1*	Chr19:24321913-24327978	6066	136

## Data Availability

No new data were created or analyzed in this study. Data sharing does not apply to this article.
